# First-in-human phase I open-label study of the anti–TIM-3 monoclonal antibody INCAGN02390 in patients with select advanced or metastatic solid tumors

**DOI:** 10.1093/oncolo/oyaf144

**Published:** 2025-07-09

**Authors:** Martin E Gutierrez, Shou-Ching Tang, John D Powderly, Ani S Balmanoukian, Paul E Hoyle, Zhiwan Dong, Lulu Cheng, Xiaohua Gong, John E Janik, Nawel Bourayou, Omid Hamid

**Affiliations:** John Theurer Cancer Center, Hackensack University Medical Center, Hackensack, NJ 07601, USA; Louisiana State University (LSU) Health Sciences Center, New Orleans, LA 70112, USA; Carolina BioOncology Institute, Huntersville, NC 28078, USA; The Angeles Clinic and Research Institute, a Cedars Sinai Affiliate, Los Angeles, CA 90025, USA; Incyte Corporation, Wilmington, DE 19803, USA; Incyte Corporation, Wilmington, DE 19803, USA; Incyte Corporation, Wilmington, DE 19803, USA; Incyte Corporation, Wilmington, DE 19803, USA; Incyte Corporation, Wilmington, DE 19803, USA; Incyte Biosciences International Sàrl, 1110 Morges, Switzerland; The Angeles Clinic and Research Institute, a Cedars Sinai Affiliate, Los Angeles, CA 90025, USA

**Keywords:** immune checkpoint inhibitors, monoclonal antibodies, carcinoma, metastases, immunotherapy, clinical trial phase I

## Abstract

**Background:**

T-cell immunoglobulin and mucin domain-containing protein-3 (TIM-3) is an immune checkpoint receptor upregulated during anti-programmed death protein-1 (PD-1)/programmed death ligand-1 (PD-L1) immunotherapy for cancer. TIM-3 blockade may improve the antitumor activity of PD-1/PD-L1inhibition. This phase 1 study evaluated INCAGN02390, a novel, fully human Fc-engineered antibody against TIM-3.

**Methods:**

INCAGN02390 was evaluated by dose escalation at 10-1600 mg infused in 14-day cycles (every 2 weeks [Q2W]) in pretreated patients with select advanced/metastatic immunogenic solid tumors. Objectives included evaluation of safety/tolerability and maximum tolerated dose (MTD) (primary), pharmacokinetics, preliminary antitumor activity, pharmacodynamics, and immunogenicity (secondary).

**Results:**

Forty patients were enrolled and treated with INCAGN02390; 60% had previously received ≥3 lines of systemic therapy. Forty-eight percent had received a prior immune checkpoint inhibitor (anti−PD-1/PD-L1 therapy, 43%; anti-cytotoxic T-lymphocyte associated protein-4 therapy, 23%). No dose-limiting toxicities (DLTs) were observed and MTD was not reached. Twelve patients (30%) had treatment-related adverse events (TRAEs), most commonly fatigue and pruritus (*n* = 3 each); 3 (8%) had grade ≥3 TRAEs. Four patients (10%) experienced sponsor-assessed irAEs. One patient (3%) achieved partial response (duration, 5.7 months) and 6 had stable disease (≥56 days in all patients, >18 months in 2 patients).

**Conclusions:**

In this heavily pretreated population, no DLTs were reported and modest efficacy was exhibited. A 400-mg Q2W dose was selected for phase II studies investigating INCAGN02390 as part of combination immunotherapies for advanced cancers.

**Implications for Practice**

Given many patients are refractory/resistant to anti–PD-1/PD-L1 immunotherapy, novel treatment options are needed. Targeting additional checkpoint receptors may in part overcome these unmet needs. In this phase I study, anti–TIM-3 antibody INCAGN02390 showed favorable tolerability at all doses tested (up to 1600 mg/day) in heavily pretreated patients with select advanced/metastatic solid tumors. The safety profile was consistent with other single TIM-3 antibodies and immune checkpoint classes. Consistent with other anti–TIM-3 molecules, antitumor effects were modest. Future studies will investigate INCAGN02390 therapeutic potential at the RP2D of 400 mg Q2W, in combination with other immunotherapies, including anti−PD-1 molecules.

## Introduction

Immune checkpoints are inhibitory pathways of the immune system that help regulate immune response and prevent autoimmunity. However, tumor cells are also able to utilize these checkpoints to downregulate immune cell activity and bypass immune surveillance.^1-3^ Immune checkpoint inhibitors (ICIs) reactivate immune cells within the tumor microenvironment and are established as standard of care in many types of cancer.^3,4^ Inhibition of the immune-suppressing programmed death protein-1 (PD-1)/programmed death ligand-1 (PD-L1) pathway is a routine approach^2^; however, many patients and tumor types either do not respond to PD-1/PD-L1 inhibitors or progress following initial tumor regression.^2,5^ Therefore, there has been intense investigation into ways to improve outcomes with ICIs, such as identification of additional immune checkpoint receptor targets to enable simultaneous targeting of multiple pathways.^6,7^ Indeed, one mechanism of resistance to PD-1/PD-L1 inhibitors is upregulation of other inhibitory receptor pathways, such as T-cell immunoglobulin and mucin domain-containing protein-3 (TIM-3) and/or lymphocyte activation gene-3 (LAG-3), which leads to further attenuation of the immune response.^8,9^ A number of ICI combinations have demonstrated improved clinical benefit compared with monotherapies. The combination of nivolumab (anti−PD-1) and ipilimumab (anti−cytotoxic T-lymphocyte associated protein-4 [CTLA-4]) improved survival compared with ipilimumab monotherapy in patients with unresectable melanoma^10-12^ and has subsequently been approved for treatment of a range of tumors.^13-16^ Recently, the combination of relatlimab (anti−LAG-3 antibody) and nivolumab showed improved progression-free survival (PFS) compared with nivolumab alone in patients with untreated metastatic or unresectable melanoma.^17^

There is increasing evidence to support TIM-3 as a therapeutic target in human cancer.^18^ TIM-3 is a transmembrane receptor that suppresses T-cell-mediated immune responses via binding to multiple ligands.^19-23^ There are 4 known TIM-3 binding ligands: galectin-9 (Gal-9), carcinoembryonic antigen-related cell-adhesion molecule-1 (CEACAM1), phosphatidylserine (PtdSer), and high mobility group protein 1 (HMGB1). Gal-9 is solely expressed on immune cells, whereas the other ligands are expressed on both immune and tumor cells.^22,24,25^ TIM-3 binding to Gal-9 induces apoptosis of T-helper (Th)1 cells increasing immune tolerance and suppressing Th1 and Th17 responses.^18,26^ TIM-3 binding to CEACAM1 also induces immune tolerance and plays a role in CD8^+^ T-cell depletion.^18,27,28^ Binding of TIM-3 to PtdSer enables recognition and clearance of apoptotic cells, NF-κβ signaling, and interleukin-2 (IL-2) secretion,^18,29,30^ and binding to HMGB-1 contributes to inhibition of innate immune response activation by impairing HMGB-1-mediated toll-like receptor signaling.^6,18,31^

TIM-3 is expressed on T cells, natural killer (NK) cells, and some antigen presenting cells,^6^ and is over expressed on most immune cells in patients with cancer.^32^ In cancer, TIM-3 expression on CD8^+^ T cells^6,33^ and NK cells^6,34^ indicates a dysfunctional, exhausted phenotype. TIM-3/PD-1 double-positive tumor-infiltrating lymphocytes (TILs) exhibit greater dysfunction than PD-1−positive TILs and represent a distinct, severely exhausted subpopulation defined by failure to proliferate and produce IL-2, tumor necrosis factor, and interferon (IFN)-γ.^7,35^ TIM-3 is expressed on TILs from a wide range of solid tumors,^18^ including hepatocellular, cervical, colorectal, and ovarian carcinomas^36^; head and neck cancers^37^; esophageal cancers^38^; renal cell carcinoma^39^; and prostate cancer.^40^ Additionally, TIM-3 expression is associated with increased risk of progression in nonsmall cell lung cancer (NSCLC),^41^ poorer outcomes in gastric cancer,^42^ and poorer overall survival and increased lymph node metastasis, tumor grade, and PD-1 expression based on a meta-analysis of >3000 patients with solid tumors.^43^

Studies in preclinical mouse tumor models show that although TIM-3 inhibitors have modest antitumor activity as monotherapy, they demonstrate considerable antitumor activity and survival when combined with PD-1/PD-L1 pathway inhibitors.^7,44^ INCAGN02390 is a novel, fully human Fc-engineered anti–TIM-3 immunoglobulin G1κ antibody (aglycosylated, N297A)^45^ (data on file, Incyte Corporation). In preclinical studies, INCAGN02390 demonstrated dose-dependent binding to TIM-3 and blockade of PtdSer–TIM-3 interactions, and elicited rapid internalization of TIM-3, which may disrupt other ligand–TIM-3 interactions^45^ (data on file, Incyte Corporation). In mouse tumor models, INCAGN02390 showed modest activity as monotherapy, but displayed cooperativity with anti–PD-1 therapy to enhance T-cell function, and increased antitumor activity in combination with an anti–PD-1 antibody^45^ (data on file, Incyte Corporation). Results of a first-in-human phase I study designed to determine safety and tolerability of INCAGN02390 in patients with advanced malignancies are reported herein.

## Methods

### Study design

INCAGN 2390-101 (ClinicalTrials.gov identifier: NCT03652077) was a phase I, open-label, nonrandomized study that evaluated safety, tolerability, pharmacokinetics (PK), and preliminary antitumor activity of INCAGN02390 in patients with advanced or metastatic solid tumors. The study was performed at 4 centers in the United States.

INCAGN02390 dose was escalated from 10 mg, infused every 2 weeks (Q2W), to 1600 mg Q2W, with a total of 7 dose levels tested (Figure S1). A 3 + 3 dose escalation design was used to determine maximum tolerated dose (MTD), maximum number of tolerated doses (MNTD), or pharmacologically active dose (PAD). In total, up to 36 evaluable patients were to be treated, with ~3-6 evaluable patients in each treatment group. Dose escalation rules are detailed in the Supplementary Methods. Maximum tolerated dose was defined as 1 dose level below that at which at least one-third of patients in a particular treatment group had dose-limiting toxicities (DLTs), monitored during a 28-day observation period). Pharmacologically active dose was defined as a dose that achieved a level of receptor occupancy considered to be biologically active for INCAGN02390. Patients received continuous treatment with INCAGN02390 on day 1 of each 14-day cycle while deriving benefit, until occurrence of unacceptable toxicity or radiographically confirmed progressive disease. Decision to continue treatment following progressive disease was per investigator’s judgment (see *Tumor imaging* section).

### Ethics approval

The study was performed in accordance with the ethical principles of the International Council for Harmonisation of Technical Requirements for Pharmaceuticals for Human Use guideline for Good Clinical Practice, the Declaration of Helsinki, and other applicable local ethical and legal requirements. The protocol (including amendments) was approved by an independent ethics committee or institutional review board before enrollment of patients at each site (Western Institutional Review Board, Puyallup, WA, USA). All patients provided written informed consent before screening.

### Patients

Eligible patients were aged ≥18 years and had locally advanced or metastatic solid tumors that progressed on, or patients were ineligible/intolerant to, treatment with available therapies likely to convey clinical benefit. Locally advanced disease could not be amenable to resection with curative intent. Eligible tumor types were immunogenic tumors for which PD-1/PD-L1 therapy is known to be beneficial (Supplementary Methods). Other immunogenic tumor types were eligible with sponsor’s approval. Patients had measurable disease based on Response Evaluation Criteria in Solid Tumors (RECIST) v1.1 and Eastern Cooperative Oncology Group performance status (ECOG PS) of 0 or 1. Fresh and/or archival tumor biopsies were required at baseline, with optional biopsies to be obtained at time of response, stable disease (SD), or disease progression.

Exclusion criteria included prior treatment with an anti–TIM-3 antibody for any indication, prior treatment with chemotherapy, targeted small-molecule therapy, anti–PD-1 pathway-targeting agent, or radiotherapy within 14 days; monoclonal antibody or other investigational agent within 28 days; immune suppressive treatment within 7 days; or live vaccine within 30 days of start of study.

### Objectives and assessments

Primary study objectives were to evaluate safety and tolerability of INCAGN02390 and to define an MTD (if reached) or PAD. Safety and tolerability were assessed by monitoring frequency, duration, and severity of adverse events (AEs) and DLTs. Dose-limiting toxicities were evaluated by investigator assessment, according to Common Terminology Criteria for Adverse Events v4.03 between day 1 of cycle 1 and study day 28 (defined in Table S1 and Supplementary Methods). Immune-related AEs (irAEs) were identified by the sponsor using predefined preferred terms that were associated with each symptom arising from each irAE. Immune relatedness of irAEs was assessed from clinical review by the sponsor.

Secondary objectives were evaluation of PK, preliminary antitumor activity of INCAGN02390, TIM-3 receptor occupancy, and immunogenicity of INCAGN02390 (defined as occurrence of specific antidrug antibodies [ADAs] to INCAGN02390). PK endpoints included evaluation of minimum (*C*_min_) and maximum (*C*_max_) observed plasma concentrations, time to *C*_max_ (*t*_max_), and area under the plasma or serum concentration–time curve (AUC) from time 0 to last measurable concentration at time *t*, analyzed after first dose of study drug and at steady state. Efficacy endpoints included assessment of objective response rate (ORR; proportion of patients with complete response or partial response [PR]), duration of response, disease control rate, and PFS, assessed by investigator using RECIST v1.1.^46^ Exploratory investigation of tumor and immune cell infiltrate biomarkers potentially predictive of INCAGN02390 pharmacological activity was an additional study objective.

### Pharmacokinetic and ADA analyses

PK and ADA analysis was performed using serum samples taken preinfusion and postinfusion (Supplementary Methods).

### Tumor imaging

Tumor response was assessed using contrast computed tomography (CT) or magnetic resonance imaging. Computed tomography component of positron emission tomography/CT could also be used with sponsor’s approval (Supplementary Methods).

### Correlative translational studies

#### TIM-3 receptor occupancy analysis

TIM-3 receptor occupancy was determined by assessing the level of INCAGN02390 binding to TIM-3 in patients infused with INCAGN02390. Flow cytometry was used to monitor total and free TIM-3 receptors on circulating monocytes and NK cells from peripheral blood. Preinfusion and end-of-infusion samples were both analyzed. Receptor occupancy was calculated as: 1 − (free median fluorescent intensity [MdFI] / total MdFI) × 100 (Supplementary Methods).

#### Plasma protein biomarker analysis and whole blood T-cell profiling

Biomarker effects of INCAGN02390 were assessed by multicolor flow cytometry. T-cell activation markers included CD38 and human leukocyte antigen–DR isotope (HLA-DR), frequency of regulatory T (Treg) cells and immune checkpoint proteins PD-1, LAG-3, and TIM-3. Circulating plasma proteins from peripheral blood were assessed using a multiplex proximity extension assay developed and performed by Olink Proteomics (Waltham, MA, USA).

### Statistical analysis

Demographics, baseline characteristics, participant disposition, and safety analyses were conducted using the safety population. Efficacy endpoints were assessed in the full analysis set, except ORR, which was assessed in the RECIST-evaluable population. The full analysis set and safety population comprised patients who had received ≥1 dose of INCAGN02390. PK/pharmacodynamics (PD) were assessed in the PK-/PD-evaluable populations, comprising patients who received ≥1 dose of INCAGN02390 and provided ≥1 postinfusion plasma sample for PK or PD analysis (Supplementary Methods).

## Results

### Patients

Forty patients were enrolled and treated between September 24, 2018, and August 19, 2021; database lock December 3, 2021. Baseline demographic and disease characteristics are summarized in Table 1. Median (range) age was 63.0 (26-90) years and 7 (18%) and 33 (83%) patients had ECOG PS of 0 and 1, respectively. Most common solid tumor types were breast cancer (15%), lung cancer (13%), and colorectal cancer (10%). All patients had metastatic disease at study entry, including metastases to lung (60%), lymph nodes (53%), and liver (35%). Almost all patients (98%) had received prior anticancer systemic therapy and 60% had received ≥3 lines of prior anticancer systemic therapy. Nineteen patients (48%) had received prior ICI therapy; 17 (43%) had received anti–PD-1/PD-L1 therapy (most commonly nivolumab, *n* = 10) and 9 (23%) had received prior CTLA-4 therapy (most commonly ipilimumab, *n* = 8). Eight patients had history of treatment with ipilimumab and a PD-1 inhibitor.

**Table 1.** Patient baseline demographics and characteristics.

Characteristic	INCAGN02390 treatment group							
	10 mg (*n *= 6)	30 mg (*n *= 6)	100 mg (*n *= 4)	200 mg (*n *= 5)	400 mg (*n *= 6)	800 mg (*n *= 6)	1600 mg (*n *= 7)	Total (*N* = 40)
Age (years), median (range)	64.0 (40-66)	62.0 (26-65)	62.0 (53-70)	63.0 (43-72)	67.0 (55-77)	64.5 (32-90)	63.0 (44-75)	63.0 (26-90)
Sex, *n* (%)								
Male	5 (83)	4 (67)	3 (75)	1 (20)	2 (33)	2 (33)	4 (57)	21 (53)
Female	1 (17)	2 (33)	1 (25)	4 (80)	4 (67)	4 (67)	3 (43)	19 (48)
Race, *n* (%)								
White	6 (100)	2 (33)	2 (50)	3 (60)	4 (67)	4 (67)	2 (29)	23 (58)
Black	0	1 (17)	1 (25)	2 (40)	1 (17)	2 (33)	2 (29)	9 (23)
Asian	0	2 (33)	0	0	1 (17)	0	2 (29)	5 (13)
Other	0	1 (17)	1 (25)	0	0	0	1 (14)	3 (8)
Tumor type, *n* (%)								
Breast cancer	1 (17)	1 (17)	0	1 (20)	1 (17)	0	2 (29)	6 (15)
Lung cancer	0	1 (17)	1 (25)	1 (20)	1 (17)	1 (17)	0	5 (13)
Colorectal cancer	1 (17)	0	1 (25)	1 (20)	0	0	1 (14)	4 (10)
Melanoma	1 (17)	0	1 (25)	0	0	1 (17)	0	3 (8)
Esophageal cancer	0	1 (17)	0	0	0	0	2 (29)	3 (8)
Cervical cancer	0	0	0	0	2 (33)	0	0	2 (5)
Cholangiocarcinoma	0	1 (17)	0	0	0	1 (17)	0	2 (5)
Sarcoma	0	1 (17)	0	0	0	1 (17)	0	2 (5)
Other solid tumors	3 (50)^a^	1 (17)^b^	1 (25)^c^	2 (40)^d^	2 (33)^e^	2 (33)^f^	2 (29)^g^	13 (33)
Number of metastatic sites at study entry, *n* (%)								
<3	2 (33)	0	3 (75)	2 (40)	4 (67)	2 (33)	2 (29)	15 (38)
≥3	4 (67)	6 (100)	1 (25)	3 (60)	2 (33)	4 (67)	5 (71)	25 (63)
ECOG PS, *n* (%)								
0	1 (17)	1 (17)	1 (25)	1 (20)	1 (17)	1 (17)	1 (14)	7 (18)
1	5 (83)	5 (83)	3 (75)	4 (80)	5 (83)	5 (83)	6 (86)	33 (83)
≥2	0	0	0	0	0	0	0	0
Types of prior therapy, *n* (%)								
Surgery	5 (83)	4 (67)	3 (75)	3 (60)	4 (67)	3 (50)	6 (86)	28 (70)
Radiotherapy	5 (83)	5 (83)	2 (50)	4 (80)	3 (50)	5 (83)	4 (57)	28 (70)
Systemic therapy	6 (100)	6 (100)	4 (100)	4 (80)	6 (100)	6 (100)	7 (100)	39 (98)
Prior anticancer systemic therapy type, *n* (%)								
Chemotherapy	6 (100)	6 (100)	4 (100)	4 (80)	5 (83)	5 (83)	7 (100)	37 (93)
Targeted therapy	1 (17)	4 (67)	2 (50)	3 (60)	4 (67)	3 (50)	5 (71)	22 (55)
Checkpoint inhibitor therapy	3 (50)	3 (50)	2 (50)	1 (20)	4 (67)	4 (67)	2 (29)	19 (48)
Other immunotherapy^h^	2 (33)	0	0	0	1 (17)	0	1 (14)	4 (10)
Number of lines of prior anticancer systemic therapy, *n* (%)								
0-1	2 (33)	0	0	1 (20)	0	1 (17)	0	4 (10)
2	2 (33)	0	3 (75)	0	1 (17)	3 (50)	3 (43)	12 (30)
≥3	2 (33)	6 (100)	1 (25)	4 (80)	5 (83)	2 (33)	4 (57)	24 (60)

Other solid tumors, all *n* = 1.

^a^Mesothelioma, osteosarcoma of right femur, gastric cancer.

^b^Pancreatic cancer.

^c^Myxoid leiomyosarcoma of the uterus.

^d^Endometrial cancer, adenoid cystic carcinoma.

^e^Extra ovarian peritoneal carcinoma, renal cell carcinoma.

^f^Right colon adenocarcinoma, salivary gland cancer.

^g^Adenocarcinoma, rectal adenocarcinoma.

^h^All patients with history of other immunotherapy also had history of treatment with a checkpoint inhibitor.

Abbreviation: ECOG PS, Eastern Cooperative Oncology Group performance status.

Patients received INCAGN02390 administered Q2W at dose levels of 10 mg (*n *= 6), 30 mg (*n *= 6), 100 mg (*n *= 4), 200 mg (*n *= 5), 400 mg (*n *= 6), 800 mg (*n *= 6), and 1600 mg (*n *= 7). All patients discontinued treatment, most commonly due to disease progression (68%) and AEs (15%) (Supplementary Results; Figure S1). No on-treatment biopsies were taken.

### Safety and tolerability

Overall, patients received a median (range) of 4.0 (1-53) infusion cycles, ranging from 2.5 (1-4) in the 10-mg treatment group to 10.5 (1-53) in the 400-mg treatment group. No DLTs and 1 sponsor-assessed infusion-related reaction (pyrexia) was observed; MTD and MNTD of INCAGN02390 were not reached. All patients had ≥1 treatment-emergent adverse event (TEAE) (Table 2); most common TEAEs were anemia (*n *= 14, 35%), back pain (*n *= 12, 30%), and fatigue (*n *= 11, 28%). There were no dose-related trends in TEAEs or consistent differences between treatment groups. Grade ≥3 TEAEs were reported in 21 patients (53%); most were grade 3 (grade 3, *n *= 17; grade 4, *n *= 3; grade 5, *n *= 1) (Table 2). Twelve patients (30%) had treatment-related TEAEs (TRAEs); those occurring in >1 patient were fatigue and pruritus (*n *= 3 each, 8%) and diarrhea, myalgia, and rash (*n *= 2 each, 5%) (Table S2). Three patients (8%) had grade ≥3 TRAEs (anemia, adrenal insufficiency, increased amylase [*n *= 1 each, 3%]). Eight patients (20%) had ≥1 TEAE leading to dose interruption, all of which resulted in delays in administering next scheduled dose. Only 2 events (adrenal insufficiency, and anemia) were considered treatment related by investigators, and dyspnea was the only TEAE leading to dose interruption that occurred in >1 patient (*n *= 2). Six patients (15%) across multiple dose levels had ≥1 TEAE leading to treatment discontinuation (10 mg Q2W, *n *= 2 [supraventricular tachycardia, sepsis, *n *= 1 each], 33%; 100 mg Q2W, *n *= 1 [acute respiratory failure], 25%; 200 mg Q2W, *n *= 1 [female genital tract fistula], 20%; 400 mg Q2W, *n *= 1 [sepsis], 17%; 1600 mg, *n *= 1 [pleural effusion], 14%); none were considered treatment related by investigators.

**Table 2.** Treatment-emergent AEs.^a^

MedDRA preferred term, *n* (%)	INCAGN02390 treatment group							Total (*N* = 40)
	10 mg (*n *= 6)	30 mg (*n *= 6)	100 mg (*n *= 4)	200 mg (*n *= 5)	400 mg (*n *= 6)	800 mg (*n *= 6)	1600 mg (*n *= 7)	
Any-grade TEAE	6 (100)	6 (100)	4 (100)	5 (100)	6 (100)	6 (100)	7 (100)	40 (100)
Anemia	1 (17)	2 (33)	0	4 (80)	0	3 (50)	4 (57)	14 (35)
Back pain	1 (17)	1 (17)	1 (25)	2 (40)	4 (67)	0	3 (43)	12 (30)
Fatigue	1 (17)	1 (17)	0	3 (60)	2 (33)	3 (50)	1 (14)	11 (28)
Decreased appetite	1 (17)	1 (17)	0	1 (20)	2 (33)	1 (17)	1 (14)	7 (18)
Nausea	1 (17)	1 (17)	0	1 (20)	2 (33)	1 (17)	1 (14)	7 (18)
Pruritus	1 (17)	1 (17)	1 (25)	1 (20)	1 (17)	2 (33)	0	7 (18)
Abdominal pain	1 (17)	0	1 (25)	0	2 (33)	0	2 (29)	6 (15)
Diarrhea	0	0	1 (25)	2 (40)	2 (33)	0	1 (14)	6 (15)
Hyponatremia	1 (17)	3 (50)	0	1 (20)	0	1 (17)	0	6 (15)
AST increased	1 (17)	2 (33)	0	1 (20)	0	1 (17)	0	5 (13)
Chills	1 (17)	1 (17)	0	1 (20)	2 (33)	0	0	5 (13)
Dehydration	1 (17)	1 (17)	1 (25)	0	2 (33)	0	0	5 (13)
Dizziness	0	1 (17)	0	0	1 (17)	2 (33)	1 (14)	5 (13)
Dyspnea	1 (17)	0	1 (25)	0	1 (17)	1 (17)	1 (14)	5 (13)
Headache	0	1 (17)	0	1 (20)	1 (17)	2 (33)	0	5 (13)
Pleural effusion	0	1 (17)	0	0	1 (17)	1 (17)	2 (29)	5 (13)
Abdominal pain upper	0	0	1 (25)	0	2 (33)	0	1 (14)	4 (10)
ALT increased	1 (17)	1 (17)	0	1 (20)	0	1 (17)	0	4 (10)
Bone pain	1 (17)	1 (17)	0	2 (40)	0	0	0	4 (10)
Constipation	0	0	1 (25)	1 (20)	0	1 (17)	1 (14)	4 (10)
Cough	0	1 (17)	0	1 (20)	1 (17)	0	1 (14)	4 (10)
Hydronephrosis	0	0	1 (25)	1 (20)	0	1 (17)	1 (14)	4 (10)
Pyrexia	1 (17)	0	0	1 (20)	1 (17)	0	1 (14)	4 (10)
Tumor pain	2 (33)	0	0	0	0	2 (33)	0	4 (10)
Grade ≥3 TEAE	4 (67)	2 (33)	2 (50)	2 (40)	4 (67)	3 (50)	4 (57)	21 (53)
Anemia	0	0	0	0	0	2 (33)	2 (29)	4 (10)
Pleural effusion	0	0	0	0	1 (17)	1 (17)	2 (29)	4 (10)
Acute respiratory failure	1 (17)	0	1 (25)	0	0	0	1 (14)	3 (8)
Back pain	1 (17)	0	0	0	2 (33)	0	0	3 (8)
Increased blood bilirubin	0	1 (17)	0	0	0	1 (17)	0	2 (5)
Dyspnea	0	0	0	0	0	1 (17)	1 (14)	2 (5)
Mental status change	1 (17)	0	0	0	0	1 (17)	0	2 (5)
Sepsis	1 (17)	0	0	0	1 (17)	0	0	2 (5)

^a^TEAEs occurring in ≥10% (any grade) and ≥5% (grade ≥3) of total patients treated with INCAGN02390 Q2W.

Abbreviations: ALT, alanine aminotransferase; AST, aspartate aminotransferase; MedDRA, Medical Dictionary for Regulatory Activities; Q2W, every 2 weeks; TEAE, treatment-emergent adverse event.

Serious TEAEs were reported in 18 patients (45%), most commonly pleural effusion (*n *= 4, 10%), acute respiratory failure (*n *= 3, 8%), and sepsis (*n *= 2, 5%). One patient had a serious grade 3 TEAE of adrenal insufficiency, which was determined to be treatment related by investigators. Four patients (10%) experienced sponsor-assessed irAEs (adrenal insufficiency [grade 3], hypothyroidism [grade 2], acute kidney injury [grade 3], dermatitis [grade 2], pruritus [grade 2], *n* = 1 each). One patient experienced 2 events of acute kidney injury and 1 event of adrenal insufficiency, all grade 3. In total, 12 patients received steroids for TEAEs including 2 patients with the previously mentioned irAEs. One patient in the 800-mg treatment group had a fatal TEAE of multiple organ dysfunction syndrome, deemed unrelated to treatment by investigator (Supplementary Results).

### Pharmacokinetics

**Figure 1.**
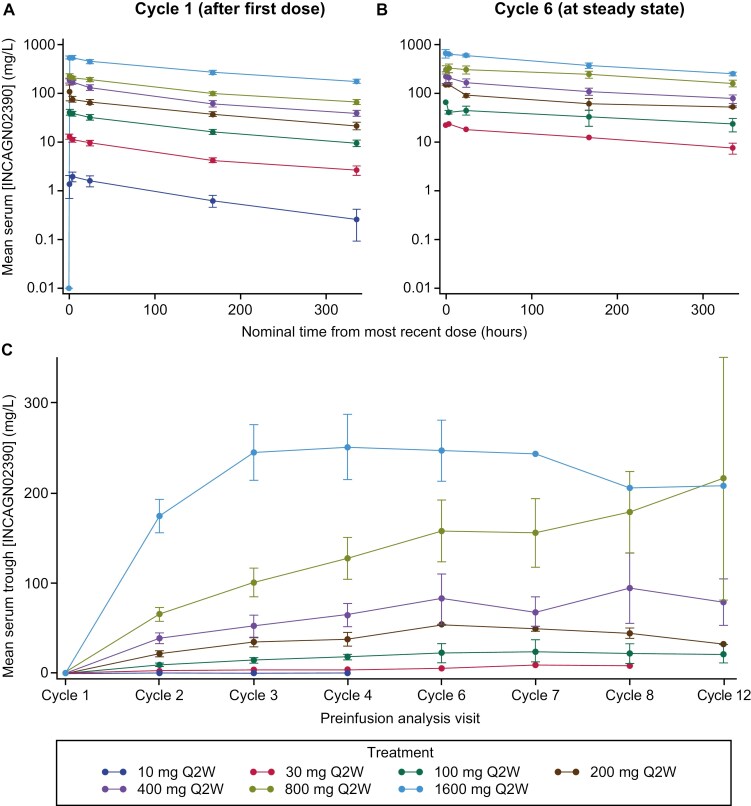
Mean (± standard error) INCAGN02390 serum concentration over time by treatment group after (A) the first dose (cycle 1), (B) at steady state (cycle 6), and (C) INCAGN02390 serum preinfusion trough concentration (*C*_min_) from cycles 1 to 12. *C*_min_, minimum observed serum concentration; Q2W, every 2 weeks.

INCAGN02390 serum concentration–time profiles at cycle 1 (post first dose) and cycle 6 (steady state) stratified by dose are shown in Figure 1; corresponding PK parameters are summarized in Table S3. *C*_max_ and AUC values were linear across most dose levels. Among all patients, mean accumulation ratios for *C*_max_ and AUC from time 0 to 336 h postdose (AUC_0–336h_) were 1.3 and 1.7, respectively, suggesting modest accumulation of INCAGN02390 at steady state. At 400 mg Q2W, after the first dose, PK data were available for 6 patients. Geometric mean *C*_max_, AUC_0–336h_, and terminal half-life at 400 mg Q2W were 172 mg/L, 1000 mg∙day/L (*n* = 5), and 6.7 days, respectively. Median *t*_max_ was 0.6 (0.5-4.3) h. At steady state, PK data were available for 3 patients. Geometric mean *C*_max_, AUC_0–336h_, and terminal half-life were 178 mg/L, 1380 mg∙day/L, and 9.3 days, respectively. Median *t*_max_ was 0.6 (0.6-0.7) h.

Among 40 evaluable patients enrolled, 33 were assessable for ADAs (ie, had ≥1 postdosing sample with reportable ADA result). Three assessable patients (9%) had a treatment-emergent ADA (10-mg, 30-mg, 400-mg treatment groups, *n* = 1 each), all of which were persistent during the study (Table S4). PK of INCAGN02390 was not impacted by ADAs in the 3 patients with treatment-emergent ADAs, based on increasing trough concentrations following multiple doses.

### Correlative translational studies

**Figure 2.**
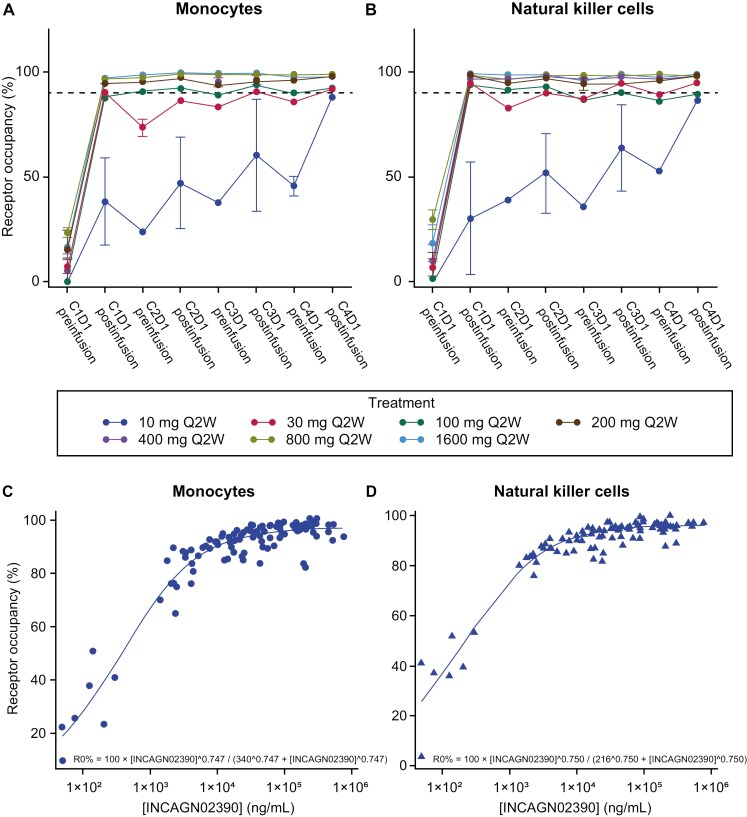
INCAGN02390 TIM-3 receptor occupancy. Average receptor occupancy at different time points preinfusion and postinfusion by treatment group on (A) monocytes and (B) NK cells by treatment group in patients receiving INCAGN02390; and relationship between serum concentration of INCAGN02390 and TIM-3 receptor occupancy on (C) monocytes and (D) NK cells. In (A) and (B), data are shown as average receptor occupancy per treatment group ± standard error at different time points preinfusion and postinfusion. 10-mg, 30-mg, 800-mg, and 1600-mg treatment groups, *n* = 6 each; 100-mg dose treatment group, *n* = 4; 200-mg and 400-mg treatment groups, *n* = 5. C, cycle; D, day; RO, receptor occupancy; TIM-3, T-cell immunoglobulin and mucin domain-containing protein-3.

TIM-3 receptor occupancy was >90% on the surface of monocytes and NK cells in peripheral blood of patients receiving INCAGN02390 doses ≥200 mg Q2W (Figure 2A and B). TIM-3 receptor occupancy on monocytes and NK cells was directly proportional to INCAGN02390 concentration; according to sigmoidal maximum effect model analyses, half-maximal effective concentration and Hill coefficient, respectively, were 340 ng/mL and 0.7 for monocytes, and 216 ng/mL and 0.8 for NK cells (Figure 2C and D). This modeling showed geometric mean INCAGN02390 trough concentration after the 400-mg Q2W dose maintained 98% of maximal effective concentration of 62.2 mg/L and 38.8 mg/L for monocytes and NK cells, respectively.

No change was seen in frequency of proliferating T cells (CD3^+^), or levels of T-cell activation markers such as CD38 and HLA-DR, or immune checkpoint proteins PD-1 and LAG-3, from cycle 1 day 1 to cycle 4 day 1. TIM-3 expression on T cells remained low across all time points. No changes were seen in frequency of either Tregs or various subsets of myeloid-derived suppressor cells (MDSCs) and NK cells. Among assessed plasma proteins, only soluble TIM-3 was significantly elevated (false discovery rate, <0.05); mean fold change of soluble TIM-3 from baseline to cycle 1 day 8 was 9.9-fold in patients receiving INCAGN02390 ≥400 mg Q2W, and 4.3-fold in those receiving <400 mg Q2W (Figure S2). IFN-inducible chemokines CXCL-9 and CXCL-10 levels remained unchanged with all doses (data on file, Incyte Corporation).

### Efficacy

One patient with adenoid cystic carcinoma of the lung receiving INCAGN02390 200 mg achieved a PR lasting 5.7 months, with PFS of 11.0 months. The patient had no history of systemic therapy or progressive disease on prior therapy. Best overall response of SD was observed in 6 patients. These patients had endometrium cancer, colorectal cancer, salivary gland cancer, renal cell carcinoma, extra ovarian peritoneal carcinoma, and right colon adenocarcinoma (all *n* = 1) (Table S5). All 6 demonstrated PFS ≥56 days (duration from start of treatment until criteria for disease progression were met; range, 56-768 days, where SD for the patient reporting 768 days was ongoing at time of censoring); 3 achieved SD >6 months. Five had history of progressive disease on prior therapy and 2 had history of ICI treatment. Overall, disease control rate (95% confidence interval [CI]) was 18% (7-33) and median PFS (95% CI) was 1.9 months (1.7-2.0) (Table 3).

**Table 3.** Summary of best overall response, objective response rate, and disease control rate by RECIST v1.1.

Parameter	INCAGN02390 treatment group							
	10 mg (*n *= 6)	30 mg (*n *= 6)	100 mg (*n *= 4)	200 mg (*n *= 5)	400 mg (*n *= 6)	800 mg (*n *= 6)	1600 mg (*n *= 7)	Total (*N* = 40)
Best overall response, *n* (%)								
Complete response	0	0	0	0	0	0	0	0
Partial response	0	0	0	1 (20)	0	0	0	1 (3)
Stable disease	0	0	0	2 (40)	2 (33)	2 (33)	0	6 (15)
>6 months	0	0	0	0	1 (17)	2 (33)	0	3 (8)
Progressive disease	2 (33)	6 (100)	4 (100)	2 (40)	3 (50)	2 (33)	5 (71)	24 (60)
Not evaluable	4 (67)	0	0	0	1 (17)	2 (33)	1 (14)	8 (20)
Missing	0	0	0	0	0	0	1 (14)	1 (3)
DCR,^a^ % (95% CI)	0 (0-46)	0 (0-46)	0 (0-60)	60 (15-95)	33 (4-78)	33 (4-78)	0 (0-41)	18 (7-33)
PFS (months), median (95% CI)	2.0 (1.9-NE)	1.6 (0.4-NE)	1.7 (1.4-NE)	11.0 (1.7-NE)	1.8 (0.8-NE)	1.9 (1.6-NE)	1.8 (0.9-2.6)	1.9 (1.7-2.0)

^a^DCR was defined as the percentage of patients with complete response, partial response, or stable disease on or after day 42.

Abbreviations: CI, confidence interval; DCR, disease control rate; NE, not evaluable; ORR, objective response rate; PFS, progression-free survival; RECIST, Response Evaluation Criteria in Solid Tumors.

## Discussion

This first-in-human, open-label, phase I study was designed to evaluate safety, tolerability, PK, and preliminary activity of INCAGN02390-treated patients with advanced or metastatic immunogenic solid tumors, and to determine recommended phase 2 dose. Maximum tolerated dose was not reached, and doses of ≥400 mg Q2W were determined to be PADs based on TIM-3 receptor occupancy. INCAGN02390 given at a dose of 400 mg Q2W was therefore chosen for further study based on receptor occupancy, as well as PK, PD, and safety data. TIM-3 receptors were >90% saturated on the surface of monocytes and NK cells in peripheral blood in patients receiving INCAGN02390 doses ≥200 mg Q2W; however, as receptor occupancy was determined using an in vitro assay, the 400-mg Q2W dose was selected to account for potentially lower drug availability within the tumor.

Overall, INCAGN02390 tolerability was favorable across all dose levels, with 6 patients experiencing a TEAE leading to INCAGN02390 discontinuation (dose levels 10 mg, 100 mg, 200 mg, 400 mg, 1600 mg), none considered treatment related. Dose interruptions were only required for 20% of patients, with only 2 patients experiencing events deemed treatment related by investigators. Fatigue and pruritus were the most common TRAEs (8% each), and only 3 patients (8%) experienced grade ≥3 TRAEs. One infusion-related reaction of pyrexia, per sponsor assessment, was reported. No dose-related TEAEs or trends suggestive of a drug effect were observed. The INCAGN02390 single-agent safety and toxicity profile was consistent with that observed with other single TIM-3 antibodies and immune checkpoint classes.^47-49^ In other phase I studies with anti–TIM-3 antibodies LY3321367 (*N* = 30) and sabatolimab (*N* = 87) no DLTs were reported, whereas among patients treated with cobolimab (*N* = 33), 1 patient (3%) reported a DLT (grade 3 increased lipase).^47-49^ As seen with INCAGN02390, fatigue was the most commonly reported TRAE, reported among 9% and 13% of patients treated with sabatolimab and cobolimab, respectively; fatigue was among the most commonly reported TRAEs with LY3321367 during dose expansion (5%).^47-49^

Treatment with INCAGN02390 monotherapy was associated with linear PK, which is generally typical for monoclonal antibodies.^50^ PK of INCAGN02390 did not appear to be impacted by treatment-emergent ADAs. However, these findings should be interpreted with caution, because of limited PK observations at later cycles and low incidence of ADAs overall.

PD analyses using peripheral blood demonstrated treatment with INCAGN02390 monotherapy up to 1600 mg Q2W did not lead to increased T-cell proliferation or elevation of other T-cell activation markers. PD-1, LAG-3, and TIM-3, as well as levels of immune subsets of MDSCs and NK cells, remained unchanged with INCAGN02390 treatment at all dose levels. We speculate this lack of observed immune response reflects the requirement for INCAGN02390 mediated TIM-3 inhibition to form part of combination therapy to impact T cells. Treatment with INCAGN02390 led to significant elevation of soluble TIM-3 levels, suggesting a stabilizing effect of the antibody on the soluble protein. The relationship between INCAGN02390 dose and the observed increase in soluble TIM-3 levels was not assessed due to limited cohort size at each dose; however, higher doses (≥400 mg) were associated with greater fold changes at cycle 1 day 8. This suggests a dose-dependent effect of INCAGN02390 on soluble TIM-3. Whether soluble TIM-3 could be a useful PD marker of INCAGN02390 treatment requires further investigation. Increases in levels of IFN-γ−inducible chemokines CXCL-9 and CXCL-10 is an established effect of treatment with PD-1–targeted ICIs^51,52^; however, treatment with INCAGN02390 did not lead to changes in these proteins, and changes in other evaluated proteins were not significant. These findings suggest that INCAGN02390 monotherapy did not increase T-cell proliferation or stimulate immune activity.

In this study, patients with advanced or metastatic solid tumors had high disease burden and were heavily pretreated, with 60% having received ≥3 lines of previous anticancer systemic therapy and 48% having received previous ICI therapy. One PR and a disease control rate of 18% were observed, with median PFS of 1.9 months. Consistent with clinical experience with other anti–TIM-3 single-agent therapies, minimal antitumor effects were observed. No responders were reported in phase I/Ib monotherapy studies with cobolimab (most common tumor types: NSCLC, melanoma),^48^ sabatolimab (most common tumor types, dose escalation phase: colorectal cancer, pancreatic cancer; dose response phase: ovarian cancer, mesothelioma),^47^ and Sym023 (patients with advanced solid tumor malignancies, metastatic cancers, and lymphomas),^53^ whereas in a phase Ia/b study with LY3321367, limited efficacy was demonstrated. In an NSCLC monotherapy expansion cohort (anti–PD-1/PD-L1 refractory patients *n* = 23, anti–PD-1/PD-L1 responders *n* = 14), 1 PR was reported, in a patient from the anti–PD-1/PD-L1 responder cohort.^49^ A synergistic effect is expected with INCAGN02390 in combination with anti–PD-1 therapy. As seen in studies with anti–LAG-3,^54^ and supported by preclinical tumor models targeting TIM-3, concomitant blockade of the PD-1/PD-L1 axis may be required for antitumor activity of anti–TIM-3 therapies.^7,44^ Improved clinical efficacy was observed when anti–TIM-3 treatments were combined with PD-1 inhibitors in 2 studies^47,48^; however, no additional benefit was observed in a study targeting TIM-3 in patients who had previously received PD-L1 antibody treatment for advanced, treatment-refractory NSCLC.^49^ Indeed, anti–TIM-3/anti–PD-1 combination therapy may provide additional benefit in immunotherapy-naive patients compared with pretreated patients and investigation of combination activity is warranted in the untreated setting.

In addition to the strong biological rationale for combining anti–TIM-3 therapy with anti–PD-1/PD-L1 therapy,^7,44,45,47,48^ there is also evidence suggesting the triple combination of anti–TIM-3, anti–PD-1/PD-L1, and anti–LAG-3 therapies may have synergistic antitumor effects compared with single therapies or dual combinations.^55^ Thus, INCAGN02390 is being investigated in combination with other ICIs in ongoing phase II studies. One phase I/II study is investigating safety and preliminary efficacy of the dual combination of INCAGN02390 and INCAGN02385 (anti–LAG-3), and the triple combination of INCAGN02390, INCAGN02385, and retifanlimab (anti–PD-1) in patients with select advanced malignancies (NCT04370704). Finally, a phase II study is investigating retifanlimab monotherapy, retifanlimab plus INCAGN02385, and the triple combination of INCAGN02390, INCAGN02385, and retifanlimab in patients with PD-L1–positive recurrent/metastatic squamous cell head and neck carcinoma (NCT05287113).

Study limitations include the small numbers of patients per dose cohort, the limited number of patients with positive immune stimulation markers, and the variability of tumor histologies in treated patients.

## Conclusion

INCAGN02390 monotherapy was associated with linear PK and no DLTs. However, INCAGN02390 monotherapy demonstrated limited efficacy in this heavily pretreated population, with 1 PR and lack of demonstrated impact on T-cell proliferation. Therapeutic potential of INCAGN02390 at a dose of 400 mg Q2W is being investigated in combination with other immunotherapies as part of combination therapy in treatment of patients with advanced cancers.

## Supplementary Material

oyaf144_suppl_Supplementary_Figures_S1-S2_Tables_S1-S5

## Data Availability

Incyte Corporation (Wilmington, DE, USA) is committed to data sharing that advances science and medicine while protecting patient privacy. Qualified external scientific researchers may request anonymized datasets owned by Incyte for the purpose of conducting legitimate scientific research. Researchers may request anonymized datasets from any interventional study (except phase I studies) for which the product and indication have been approved on or after January 1, 2020, in at least 1 major market (eg, US, EU, JPN). Data will be available for request after the primary publication or 2 years after the study has ended. Information on Incyte’s clinical trial data sharing policy and instructions for submitting clinical trial data requests are available at: https://www.incyte.com/Portals/0/Assets/Compliance%20and%20Transparency/clinical-trial-data-sharing.pdf?ver=2020-05-21-132838-960.
