# Supplementary material for: First-in-human phase I open-label study of the anti–TIM-3 monoclonal antibody INCAGN02390 in patients with select advanced or metastatic solid tumors
Source: Oncologist. 2025 Jul 9;30(7):oyaf144. doi: 10.1093/oncolo/oyaf144 (PMC12238946; doi:10.1093/oncolo/oyaf144)

**SUPPLEMENTARY MATERIAL**

**First-in-Human Phase I Open-Label Study of the Anti–TIM-3 Monoclonal Antibody INCAGN02390 in Patients With Select Advanced or Metastatic Solid Tumors**

Martin E. Gutierrez^1^, Shou-Ching Tang^2^, John D. Powderly II^3^, Ani S. Balmanoukian^4^, Paul E. Hoyle^5^, Zhiwan Dong^5^, Lulu Cheng^5^, Xiaohua Gong^5^, John E. Janik^5^, Nawel Bourayou^6^, Omid Hamid*^,4^

^1^John Theurer Cancer Center, Hackensack University Medical Center, Hackensack, NJ, USA

^2^Louisiana State University (LSU) Health Sciences Center, New Orleans, LA, USA

^3^Carolina BioOncology Institute, Huntersville, NC, USA

^4^The Angeles Clinic and Research Institute, a Cedars Sinai Affiliate, Los Angeles, CA, USA

^5^Incyte Corporation, Wilmington, DE, USA

^6^Incyte Biosciences International Sàrl, Morges, Switzerland

**Running title:** Anti–TIM-3 mAB INCAGN02390 for advanced malignancies

***Corresponding author:** Omid Hamid, MD, The Angeles Clinic and Research Institute, a Cedars Sinai Affiliate, Los Angeles, CA, 90025, USA. Phone: (310) 294-0438. Email: [ohamid@theangelesclinic.org](mailto:ohamid@theangelesclinic.org)

**Table of** **Contents**

[Supplementary Methods 3](#_Toc193985228)

[Eligible Tumor Types 3](#_Toc193985229)

[Safety 3](#_Toc193985230)

[Rationale for Dose 3](#_Toc193985231)

[Dose Escalation Rules 4](#_Toc193985232)

[Criteria for Permanent Discontinuation of INCAGN02390 5](#_Toc193985233)

[PK and Antidrug antibody (ADA) Analyses 5](#_Toc193985234)

[Tumor Imaging 7](#_Toc193985235)

[TIM-3 Receptor Occupancy Analysis 8](#_Toc193985236)

[Statistical Methods 8](#_Toc193985237)

[Supplementary Results 9](#_Toc193985238)

[Safety Outcomes 9](#_Toc193985239)

[Discontinuations Due To Death 9](#_Toc193985240)

[Fatal TEAEs 10](#_Toc193985241)

[Supplementary Table S1. Definition of DLT (28-day DLT observation period). 11](#_Toc193985242)

[Supplementary Table S2. Treatment-related TEAEs occurring in >1 patient treated with INCAGN02390. 13](#_Toc193985243)

[Supplementary Table S3. Pharmacokinetic parameters of INCAGN02390 after first dose and at steady state by treatment group. 14](#_Toc193985244)

[Supplementary Table S4. Summary of immunogenicity for INCAGN02390. 17](#_Toc193985245)

[Supplementary Table S5. Select characteristics of patients achieving PR or SD following treatment with INCAGN02390. 18](#_Toc193985246)

[Supplementary Figure S1. Patient disposition 20](#_Toc193985247)

[Supplementary Figure S2. Soluble TIM-3 in the plasma of patients receiving INCAGN02390 21](#_Toc193985248)

# Supplementary Methods

## Eligible Tumor Types

Eligible immunogenic tumor types included locally advanced or metastatic cervical cancer, gastric cancer (including stomach and gastroesophageal junction), esophageal cancer, hepatocellular carcinoma, melanoma (uveal melanoma excluded), Merkel cell carcinoma, mesothelioma, microsatellite instability-high/deficient mismatch repair tumors, non-small cell lung cancer, ovarian cancer, squamous cell carcinoma of the head and neck, small cell lung cancer, renal cell carcinoma, triple-negative breast cancer, and urothelial carcinoma, or alternative immunogenic tumor types with medical monitor approval.

## Safety

Dose-limiting toxicities (DLTs) included nonhematologic, hematologic, and immune-related toxicity events, as well as events related to maximum tolerated dose (MTD), maximum number of tolerated doses, and deaths not clearly due to underlying disease or extraneous cause. Treatment-emergent adverse events (TEAEs) were summarized using Medical Dictionary for Regulatory Activities (version 24.0) preferred terms, and severity was graded based on National Cancer Institute Common Terminology Criteria for Adverse Events v4.03.

## Rationale for Dose

The safe starting dose (SSD) of 10 mg Q2W (or approximately 0.14 mg/kg in a 70 kg person) was based on a minimum anticipated biological effect level derived from an in vitro T-cell activation assay measuring cytokine release. This assay was considered to be the most relevant assay for consideration of pharmacology and safety and the derived SSD was shown to provide a sufficient margin of safety in a non-clinical toxicology study in cynomolgus monkeys (data on file, Incyte Corporation). The SSD was derived from the average antibody concentration required for an individual to experience 50% of the maximum effect (EC_50_) determined using the invitro T-cell activation assay (1.5μg/mL), and was converted to a projected dose of 12 mg (or 0.17 mg/kg in a 70 kg person) using a calculated average steady state serum concentration/dose from the 100 mg/kg dose in the 4-week toxicity study in cynomolgus monkeys (data on file, Incyte Corporation). Dose escalations were based on the need to distinguish pharmacokinetic (PK) parameters with a 3-fold increase in dose for each dose level increase. The maximum dose was based on the expectation that a 10 mg/kg dose level would be adequate to saturate receptor binding throughout the dosing interval.

## Dose Escalation Rules

The dose escalation study used a 3 + 3 design to evaluate the MTD or pharmacologically active dose of INCAGN02390. Patients were assigned to treatment groups (*n* ≥ 3), and dose levels of 10, 30, 100, 200, 400, 800, and 1600 mg every 2 weeks were planned to be tested. There was a 48-hour waiting period between dosing of the first and second patient of each treatment group, and the first 3 patients of a treatment group were observed for DLTs (Supplementary Table S1) for 28 days before the next treatment group began enrollment. Patients in treatment group 1 received INCAGN02390 10 mg on day 1 of a 14-day cycle. The dose was escalated (and enrollment for the next treatment group began) if none of the first 3 evaluable patients had a DLT. If 1 of the first 3 patients had a DLT, then the treatment group would be expanded to include an additional 3 patients, and if no further DLT occurred in those 3 patients, the dose would be escalated. If a DLT occurred in ≥33% of an expanded treatment group, the MTD would be deemed to be exceeded, and the previous dose level would be considered the MTD. If the MTD was exceeded with the starting dose, then a dose of 3 mg would be considered and/or alternate dosing schedules for the 10-mg dose would be explored. Throughout the treatment period, if >33% of ≥6 patients experienced a grade ≥3 toxicity related to study drug after completing ≥4 cycles, the maximum number of tolerated doses and required responses would be discussed by the sponsor and investigators. The study would end when all patients had completed the last follow-up visit or had discontinued study drug and completed follow-up assessments, or when no more than 5 patients remained on study drug for ≥6 months.

## Criteria for Permanent Discontinuation of INCAGN02390

Patients with unacceptable toxicity not caused by the underlying disease were permanently discontinued from the study. Unacceptable toxicity was defined as occurrence of an adverse event related to INCAGN02390 that (per judgment of the investigator or the sponsor’s medical monitor) compromises the patient’s ability to continue study-specific procedures or makes continuation of the study not in the patient’s best interest; or a persistent adverse event requiring a delay of INCAGN02390 therapy for >28 days, unless a greater delay is approved by the sponsor.

## PK and Antidrug antibody (ADA) Analyses

PK and ADA analysis was performed using serum samples taken pre-and post-infusion on day 1 of cycles 1, 2, 3, 6, 8, and 12. Further serum samples were collected for PK analysis on days 1 (±10 minutes post infusion and 4 hours post infusion), 2 (24 hours post infusion), and 8 (untimed) of cycle 1; day 1 of cycle 4 (pre infusion); days 1 (±10 minutes post infusion and 4 hours post infusion), 2 (24 hours post infusion), and 8 (untimed) of cycle 6; and day 1 of cycle 7 (pre infusion). Serum samples were also collected for PK and ADA analysis at the first posttreatment safety follow-up visit at 30 days after the end-of-treatment visit.

INCAGN02390 serum concentration was measured using a validated electrochemiluminescence (ECL) method (TM-JOD-0001, Immunologix Laboratories, Tampa, FL, USA), with standard Meso Scale Discovery (MSD) plates coated with an anti-idiotypic antibody against INCAGN02390 (Green Mountain Antibodies, Burlington, VT, USA). Bound INCAGN02390 was detected with biotinylated anti-INCAGN02390 (Bt Detector Antibody, Immunologix Laboratories). Data were analyzed by standard noncompartmental analysis methods using Phoenix WinNonlin v8.3.4 (Certara USA Inc, Princeton, NJ, USA). Preinfusion PK samples were assigned time 0. A validated ECL assay (TM-JOD-0002, Immunologix Laboratories) was used to detect, confirm, and titrate INCAGN02390 ADAs in human serum samples.

For the screening assay, patient samples and controls were added to transfer plates (24 μL/well) and acidified by 1:5 dilution with 100 mM glycine, pH 2.3. Plates were covered and incubated at ambient temperature for 10-20 minutes, with shaking at 450 rpm. Samples were neutralized by addition of an equal volume of neutralization buffer (50% Tris buffer [1M, pH 7.5]: 50% assay diluent [1% bovine serum albumin in 1 × phosphate-buffered saline]) containing 2 μg/mL biotin-INCAGN02390 (Bt-INCAGN02390, Immunologix Laboratories) and 2 μg/mL SulfoTag-INCAGN02390 (Ru-INCAGN02390, Immunologix Laboratories), and incubated at ambient temperature for 50-70 minutes, with shaking at 450 rpm. 100 μL neutralized samples and controls were transferred to preblocked streptavidin-coated MSD plates and incubated for 50-70 minutes at ambient temperature, with shaking at 450 rpm. After washing, 150 μL 2 × MSD read buffer was added and the resultant signal was read using an MSD Sector Imager plate reader to determine the presence of ADAs. Presence of ADAs was confirmed by analysis of samples and controls as above, with and without excess INCAGN02390 (50 mg/mL) added to the biotin- and SulfoTag-INCAGN02390 containing neutralization buffer. Samples showing a specified (≥35%) reduction in signal in the presence of excess INCAGN02390 were designated ADA positive.

## Tumor Imaging

Presence of measurable disease at study entry was confirmed by initial tumor imaging ≤28 days before the patient received their first dose of INCAGN02390. During the study, imaging was performed 8 weeks after the first dose of INCAGN02390 and then every 8 weeks for the first 12 months, and then every 12 weeks until disease progression was determined. Response to treatment was confirmed by repeat radiographic assessment, per Response Evaluation Criteria in Solid Tumors (RECIST) v1.1 ≥4 weeks after first documentation of response or at the next scheduled scan, as per clinical indication. This imaging schedule was followed until the earliest occurrence of death, documented disease progression, initiation of a new anticancer treatment, withdrawal of consent, or the end of the study. In patients showing radiological evidence of disease progression per RECIST v1.1, and who were considered clinically stable by investigators (those with an with absence of symptoms and signs indicating clinically significant disease progression, no decline in Eastern Cooperative Oncology Group performance status, and no requirement for intensified management, including increased analgesia, radiation, or palliative care), study treatment could continue pending investigator-assessed confirmation per Immune Response Evaluation Criteria in Solid Tumors (iRECIST) by repeat imaging >4 and ≤8 weeks after initial indication. Clinically unstable patients were discontinued from the study following central verification of initial radiological evidence of disease progression, without requirement of repeat imaging.

## TIM-3 Receptor Occupancy Analysis

Briefly, whole blood collected from patients was incubated with Fc block and stained with antibodies against extracellular markers, including CD45, CD14, CD19, CD3, and CD56, and 3 antibodies against T-cell immunoglobulin and mucin domain-containing protein-3 (TIM-3). Unconjugated INCAGN02390 was used to block TIM-3 receptors, INCAGN02390-PE was used as a competitive antibody to detect unbound cell surface TIM-3 receptors, and commercially obtained TIM-3 (7D3 clone) AF647 (BD Biosciences, Franklin Lakes, NJ, USA) was used as a noncompetitive TIM-3 antibody to quantify total TIM-3 cell surface receptors.

## Statistical Methods

No formal statistical tests were performed in this exploratory study and all confidence intervals (CIs) were reported to 95%. Safety measures, including treatment-emergent adverse events, laboratory values, vital signs, and electrocardiograms, were summarized with descriptive statistics. Objective response rate and its 95% CI were presented by treatment group. For the RECIST-evaluable population, progression-free survival (PFS) was analyzed using the Kaplan-Meier method; median PFS was estimated with 95% CIs for each treatment group. Treatment effects on biomarker expression were assessed by paired *t* test comparing baseline (at cycle 1 day 1) to on-treatment values at cycle 1 day 8 and/or cycle 2 day 1. Pharmacokinetic data were summarized with descriptive statistics. Dose proportionality was evaluated by a power function model, with the estimated exponent (and 90% CI) on dose reported. The number and percentage (where applicable) of patients determined to be ADA evaluable, ADA assessable, and ADA negative versus treatment-emergent ADA positive and persistent treatment-emergent ADA positive were summarized with descriptive statistics.

# Supplementary Results

## Safety Outcomes

## Discontinuations Due To Death

One patient (age 75 years, male), with esophageal cancer and extensive baseline metastasis to the liver, lung, skin or subcutaneous tissue, adrenal, renal, and gluteus muscle and spleen, received 1 cycle of INCAGN02390 1600 mg. On day 8 they were admitted with shortness of breath and treated/managed for acute hypoxic respiratory failure and pleural effusion, recovering on day 18. Study treatment continued unchanged in response to the serious AEs.

A second patient (age 63, male) with esophageal cancer and baseline esophageal right mass metastasis received 3 INCAGN02390 1600 mg infusions. The patient was admitted with hemoptysis on day 5 and treated with dextromethorphan-guaifenesin, budesonide, and codeine. A second episode of hemoptysis was reported on day 10, with the patient readmitted. Heavy staphylococcus aureus was detected by lower respiratory culture and treated with amoxicillin-clavulanate and piperacillin-tazobactam. The patient was readmitted on day 16 with right lower lobe pneumonia and treated with a sequence of antibiotic regimens before hospital discharge. The final dose of INCAGN02390 was continued with dose unchanged. The patient died on day 40 due to progressive disease. Neither the hemoptysis or pneumonia were considered to be caused by INCAGN02390 per investigator assessment.

## Fatal TEAEs

One patient in the 800-mg treatment group had a fatal TEAE of multiple organ dysfunction syndrome, deemed unrelated to treatment by investigator. This patient (age 33, female) with cholangiocarcinoma and baseline breast, liver, lung, and abdomen metastasis, first received INCAGN02390 800 mg on Day 1 and ended dosing with INCAGN02390 800 mg on Day 29, discontinuing treatment on Day 43 due to progressive disease after a total of 3 INCAGN02390 infusions. The patient experienced plural effusion on day 47 and was admitted to hospital for shortness of breath. The patient was obtunded on day 48 and was emergently intubated. The patient experienced multiple organ failure, onset day 48, and died on day 49 due to multiorgan failure. No immune-related AEs, including pneumonitis, and adrenal insufficiency, were reported for the patient. Per the investigator, the hepatic encephalopathy and pleural effusion were related to disease progression. Multiple organ dysfunction was considered not treatment related per the investigator assessment.

# Supplementary Table S1. Definition of DLT (28-day DLT observation period).

| **Nonhematologic toxicity** |
| --- |
| - Any liver function abnormalities that meet the definition of Hy's law^a^ - Encephalopathy of any grade - Any-grade ≥3 nonhematologic toxicity, except the following:   - Transient (≤72 hours) abnormal laboratory values without associated clinically significant signs or symptoms   - Nausea, vomiting, and diarrhea adequately controlled with supportive care within 48 hours   - Changes in cholesterol and triglycerides   - Asymptomatic changes in lipid profiles   - Asymptomatic changes in amylase and lipase   - Singular or nonfasting elevations in blood glucose (ie, blood glucose excursions will be considered toxicities if fasting blood glucose is elevated on 2 separate occasions) |
| **Hematologic toxicity** |
| - Grade 3 thrombocytopenia with clinically significant bleeding (ie, requires hospitalization, transfusion of blood products, or other urgent medical intervention) - Grade 4 thrombocytopenia - Grade ≥3 febrile neutropenia (absolute neutrophil count <1.0 × 10^9^/L and fever >101°F/38.3°C) - Grade 4 neutropenia that does not recover to grade ≤2 in ≤3 days after interrupting study drug - Grade 4 anemia not explained by underlying disease or some other concomitant disorder |
| **Immune-related toxicity** |
| - Grade ≥2 ocular irAEs - Grade 3 irAEs that do not improve to baseline or at least grade 1 in <5 days with appropriate care or with corticosteroid therapy - Grade 4 irAEs, regardless of duration |
| **General** |
| - Any death not clearly due to the underlying disease or extraneous causes - Inability to receive the planned number of doses within the 28-day DLT period due to toxicity, regardless of grade |
| **MTD** |
| - In part 1 of the study, the MTD will be defined as 1 dose level below that at which at least one-third of patients in a particular treatment group have DLTs - In part 2 of the study, toxicities will continue to be monitored. If the cumulative incidence of DLTs occurs in ≥33% of patients with solid tumors after 6 patients have been observed for ≥28 days, further enrollment may be interrupted, and the investigators and sponsor will meet and reassess the MTD |
| **MNTD** |
| - For each dose and schedule explored, if >33% of patients (minimum of 6 patients) experience a grade ≥3 toxicity related to study drug after completing ≥4 cycles, the sponsor and investigators will discuss possible MNTD and actions to be taken based on all available safety data. All AEs, regardless of the time of occurrence on study, may be considered in DLT determination decisions |

^a^Hy's law is defined as (1) ALT or AST elevation >3 × ULN, (2) total bilirubin >2 × ULN without initial findings of cholestasis (elevated serum alkaline phosphatase), and (3) no other apparent possible causes of aminotransferase elevation and hyperbilirubinemia, including, but not limited to, viral hepatitis, pre-existing chronic or acute liver disease, or the administration of other drug(s) known to be hepatotoxic.

Abbreviations: AE, adverse event; ALT, alanine aminotransferase; AST, aspartate aminotransferase; DLT, dose-limiting toxicity; irAE, immune-related adverse event; MTD, maximum tolerated dose; MNTD, maximum number of tolerated doses; ULN, upper limit of normal.

## **Supplementary Table S2.** Treatment-related TEAEs occurring in >1 patient treated with INCAGN02390.

| **MedDRA preferred term, *n* (%)** | **INCAGN02390 treatment group** | | | | | | | **Total  (*N* = 40)** |
| --- | --- | --- | --- | --- | --- | --- | --- | --- |
|  | **10 mg Q2W (*n* = 6)** | **30 mg Q2W (*n* = 6)** | **100 mg Q2W (*n* = 4)** | **200 mg Q2W  (*n* = 5)** | **400 mg Q2W  (*n* = 6)** | **800 mg Q2W  (*n* = 6)** | **1600 mg Q2W (*n* = 7)** |  |
| Any-grade treatment-related TEAE | 2 (33) | 0 | 2 (50) | 2 (40) | 1 (17) | 2 (33) | 3 (43) | 12 (30) |
| Fatigue | 1 (17) | 0 | 0 | 0 | 0 | 1 (17) | 1 (14) | 3 (8) |
| Pruritus | 1 (17) | 0 | 1 (25) | 0 | 0 | 1 (17) | 0 | 3 (8) |
| Diarrhea | 0 | 0 | 1 (25) | 1 (20) | 0 | 0 | 0 | 2 (5) |
| Myalgia | 1 (17) | 0 | 0 | 0 | 1 (17) | 0 | 0 | 2 (5) |
| Rash | 1 (17) | 0 | 1 (25) | 0 | 0 | 0 | 0 | 2 (5) |

Abbreviations: MedDRA, Medical Dictionary for Regulatory Activities; Q2W, every 2 weeks; TEAE, treatment-emergent adverse event.

Supplementary Table S3. Pharmacokinetic parameters of INCAGN02390 after first dose and at steady state by treatment group.

| **INCAGN02390 treatment group** |  | | | | | | |
| --- | --- | --- | --- | --- | --- | --- | --- |
|  | **C_max_ (mg/L)** | **t_max_ (hours)** | **C_min_ (mg/L)** | **AUC_0–336h_ (mg∙day/L)** | **t_1/2_ (days)** | **CL (L/day)** | **V_z_ (L)** |
| Pharmacokinetic parameters of INCAGN02390 after first dose^a^ | | | | | | | |
| 10 mg (*n* = 6) | 2.0 ± 1.2  (1.7) | 4.1 (0.5-4.8) | – | 11.4 ± 9.1 (9.3) [*n* = 4] | 4.4 ± 1.5 (4.1) [*n* = 4] | 1.1 ± 0.6 (1.0) [*n* = 4] | 6.6 ± 4.1 (5.7) [*n* = 4] |
| 30 mg (*n* = 6) | 12.8 ± 4.1 (12.2) | 0.5 (0.5-4.8) | – | 70.5 ± 18.2 (68.4) | 6.3 ± 2.1 (6.0) | 0.4 ± 0.1 (0.3) | 3.1 ± 1.2 (2.9) |
| 100 mg (*n* = 4) | 40.5 ± 10.4  (39.3) | 0.7 (0.6-4.7) | – | 258 ± 65.2 (251) | 6.9 ± 0.5 (6.9) | 0.3 ± 0.1 (0.3) | 3.0 ± 0.8 (2.9) |
| 200 mg (*n* = 5) | 108 ± 86.9  (89.0) | 0.6 (0.6-4.5) | – | 556 ± 115 (548) | 8.6 ± 1.3 (8.6) [*n* = 4] | 0.2 ± 0.1 (0.2) [*n* = 4] | 2.9 ± 0.6 (2.9) [*n* = 4] |
| 400 mg (*n* = 6) | 177 ± 39.9  (172) | 0.6 (0.5-4.3) | – | 1030 ± 261 (1000) [*n* = 5] | 7.0 ± 2.6 (6.7) [*n* = 5] | 0.3 ± 0.1 (0.30) [*n* = 5] | 3.0 ± 0.8 (2.9) [*n* = 5] |
| 800 mg (*n* = 6) | 226 ± 58.5  (219) | 0.5 (0.4-24.0) | – | 1550 ± 345 (1510) [*n* = 5] | 7.8 ± 2.0 (7.5) [*n* = 5] | 0.4 ± 0.1 (0.4) [*n* = 5] | 4.1 ± 1.1 (4.0) [*n* = 5] |
| 1600 mg (*n* = 7) | 573 ± 106  (565) | 0.6 (0.5-24.9) | – | 4110 ± 877 (4030) [*n* = 5] | 10.9 ± 5.2 (10.2) [*n* = 5] | 0.3 ± 0.1 (0.2) [*n* = 5] | 3.6 ± 1.1 (3.5) [*n* = 5] |
| All (*N* = 40) | – | 0.6 (0.4-24.9) | – | – | 7.5 ± 3.1 (6.9) [*n* = 33] | 0.4 ± 0.3 (0.3) [*n* = 33] | 3.7 ± 1.9 (3.4) [*n* = 33] |
| Exponent on dose (90% CI) | 1.077  (1.0-1.2) | – | – | 1.1  (1.0-1.2) | – | – | – |
| Pharmacokinetic parameters of INCAGN02390 at steady state^a^ | | | | | | | |
| 30 mg (*n* = 1) | 22.9 | 4.0 | 5.7 | 184 | 14.3 | 0.2 | 3.4 |
| 100 mg (*n* = 2) | 64.0, 37.2 | 0.6, 4.7 | 33.0, 0.3 | 611, 290 | 22.4, 7.4 | 0.2, 0.3 | 5.3, 3.7 |
| 200 mg (*n* = 2) | 143, 162 | 0.7, 4.5 | 44.1, 54.0 | 816, 1090 | 10.7, 14.3 | 0.2, 0.2 | 3.8, 3.8 |
| 400 mg (*n* = 3) | 205 ± 140 (178) | 0.6 (0.6- 0.7) | 69.7 ± 55.7 (57.4) | 1470 ± 698 (1380) | 9.4 ± 1.7 (9.3) | 0.3 ± 0.1 (0.3) | 4.1 ± 1.6 (3.9) |
| 800 mg (*n* = 4) | 330 ± 129 (309) | 4.3 (4.2-23.6) | 157 ± 70.2 (145) | 3620 ± 917 (3540) [*n* = 3] | 22.6, 20.5 | 0.2 ± 0.1 (0.2) [*n* = 3] | 7.1, 5.2 |
| 1600 mg (*n* = 2) | 580, 769 | 4.5, 0.6 | 213, 281 | 5030, 6260 | 11.1, 11.9 | 0.3, 0.3 | 5.1, 4.4 |
| All (*n* = 14) | – | 4.1 (0.6-23.6) | – | – | 13.6 ± 5.4 (12.7) [*n* = 12] | 0.3 ± 0.1  (0.2) [*n* = 13] | 4.5 ± 1.3  (4.4) [*n* = 12] |
| Exponent on dose (90% CI) | 0.8  (0.7-1.0) | – | – | 0.9  (0.8-1.0) | – | – | – |

^a^Values are shown as mean ± standard deviation (geometric mean) [*n* = number of observations], except for t_max_, which is shown as median (range). Individual patient values are provided when *n* < 3.

Abbreviations: AUC_0-336_, area under the concentration–time curve from time 0 to 336 hours post dose; CI, confidence interval; CL, clearance; C_max_, maximum observed plasma concentration; C_min_, minimum observed plasma concentration; t_1/2_, apparent terminal-phase disposition half-life; t_max_, time to maximum concentration; V_z_, volume of distribution.

## **Supplementary Table S4.** Summary of immunogenicity for INCAGN02390.

| **INCAGN02390 treatment group,  *n* (%)** | **Total patients with  ADA-assessable samples^a^** | **Patients with treatment-emergent**  **positive immunogenicity status,^b^** |
| --- | --- | --- |
| 10 mg | 4 (67) | 1 (17) |
| 30 mg | 4 (67) | 1 (17) |
| 100 mg | 4 (100) | 0 |
| 200 mg | 5 (100) | 0 |
| 400 mg | 5 (83) | 1 (17) |
| 800 mg | 5 (83) | 0 |
| 1600 mg | 6 (86) | 0 |
| All | 33 (83) | 3 (7.5) |

^a^ADA-assessable patients was defined as those with ≥1 postinfusion sample with a reportable ADA result, either negative or positive. ^b^Treatment-emergent ADA positive refers to patients who were negative at baseline but had ≥1 positive ADA sample post baseline.

Abbreviation: ADA, antidrug antibody.

## **Supplementary Table S5.** Select characteristics of patients achieving PR or SD following treatment with INCAGN02390.

| Parameter | Patient 1 | Patient 2 | Patient 3 | Patient 4 | Patient 5 | Patient 6 | Patient 7 |
| --- | --- | --- | --- | --- | --- | --- | --- |
| INCAGN02390 treatment group | 200 mg Q2W | 200 mg Q2W | 200 mg Q2W | 400 mg Q2W | 400 mg Q2W | 800 mg Q2W | 800 mg Q2W |
| Tumor type | Other: adenoid cystic carcinoma | Endometrium | Colorectal | Renal cell carcinoma | Other: extra ovarian peritoneal carcinoma | Other: colon adenocarcinoma | Salivary gland |
| Prior systemic therapy | None | Hormonal therapy, chemotherapy, targeted therapy | Targeted therapy, chemotherapy | Targeted therapy, ICI (nivolumab), ICI (2 x investigational drug monotherapies) | Chemotherapy, targeted therapy | Chemotherapy, targeted therapy | Hormonal therapy, targeted therapy, chemotherapy, investigational therapy, ICI (investigational drug) |
| Prior radiotherapy | Y | Y | N | N | N | Y | Y |
| Prior Surgery | Y | N | Y | Y | Y | Y | N |
| History of PD on prior treatment | N | Y | Y | Y | Y | N | Y |
| BOR | PR | SD | SD | SD | SD | SD | SD |
| Duration of response (months) | 5.7 | / | / | / | / | / | / |
| PFS duration^a^ (months) | 11.0 | 5.4 | 1.9 | 5.4 | 25.4 | 6.1 | 18.3 |

^a^Associated with BOR.

Abbreviations: BOR, best overall response; ICI, immune checkpoint inhibitor; Q2W, every 2 weeks; PD, progressive disease; PFS, progression-free survival; PR, partial response; SD, stable disease.

Supplementary Figure S1. Patient disposition (*N* = 40); all doses Q2W. Q2W, every 2 weeks.

**
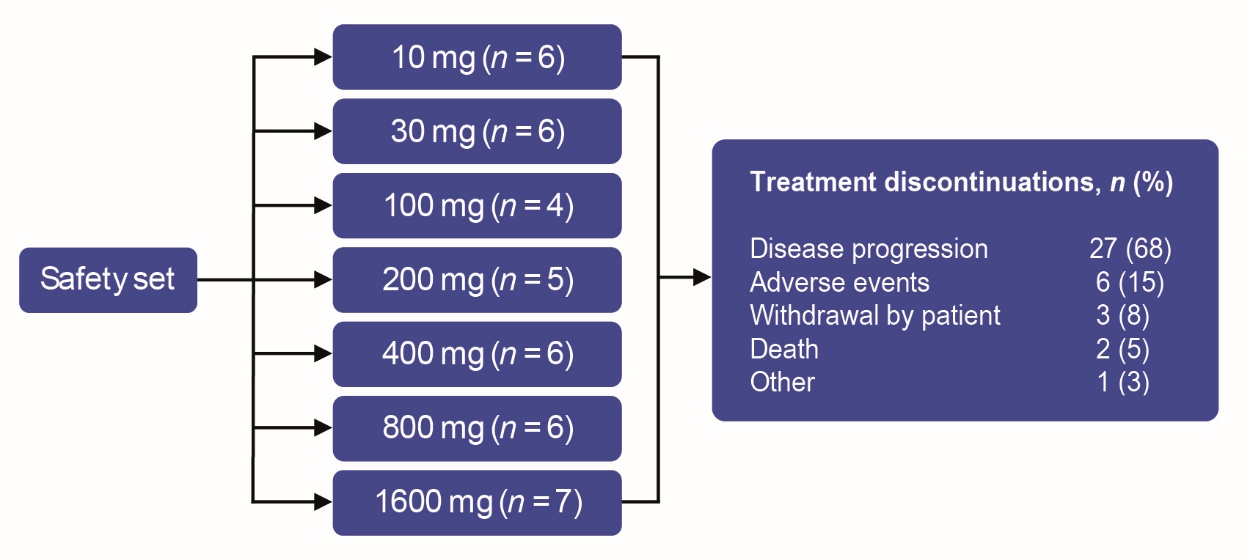
**

Supplementary Figure S2. Soluble TIM-3 in the plasma of patients receiving INCAGN02390. Line graphs show changes in soluble TIM-3 levels over time. Patients were stratified into 2 groups; patients treated with INCAGN02390 <400 mg Q2W, and patients treated with INCAGN02390 ≥400 mg Q2W. Paired *t* tests were used to calculate *P* values and determine significance (*P* < .05) of the elevation in soluble TIM-3 from C1D1 to C1D8. C, cycle; D, day; Q2W, every 2 weeks; TIM-3, T-cell immunoglobulin and mucin domain-containing protein-3.


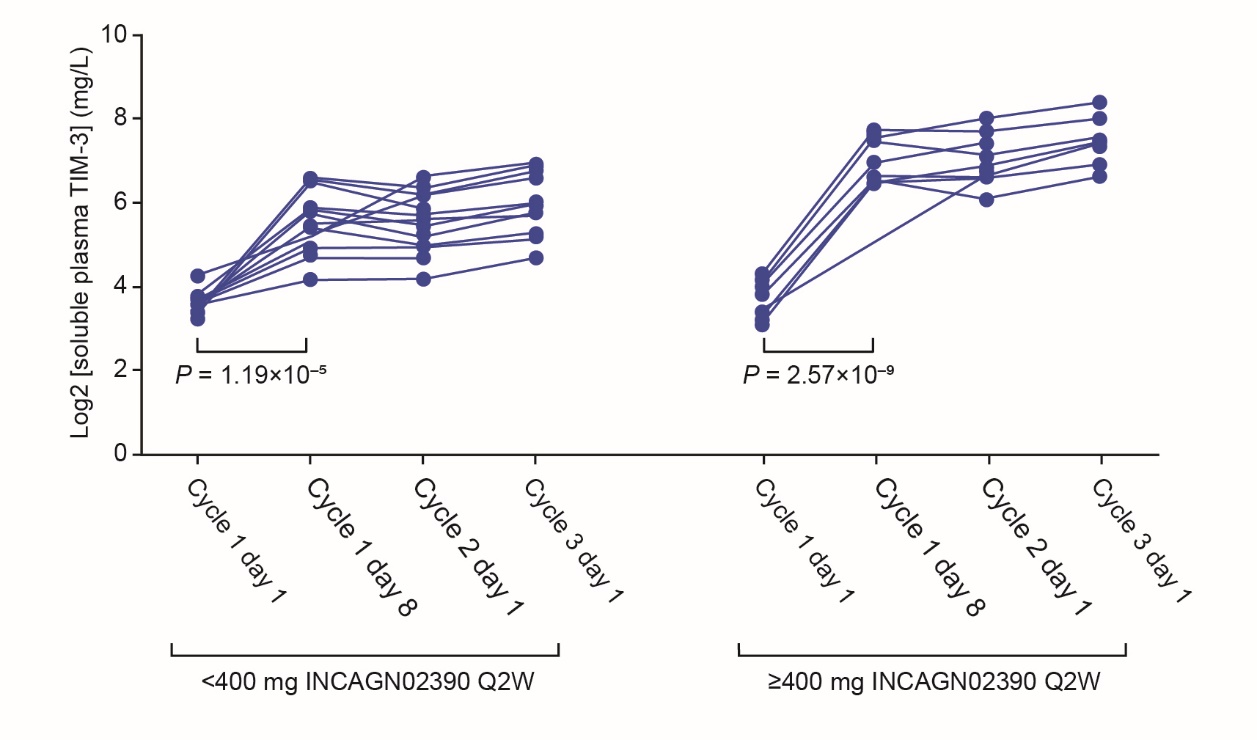

Supplement: oyaf144_suppl_Supplementary_Figures_S1-S2_Tables_S1-S5 [file oyaf144_suppl_supplementary_figures_s1-s2_tables_s1-s5.docx]
